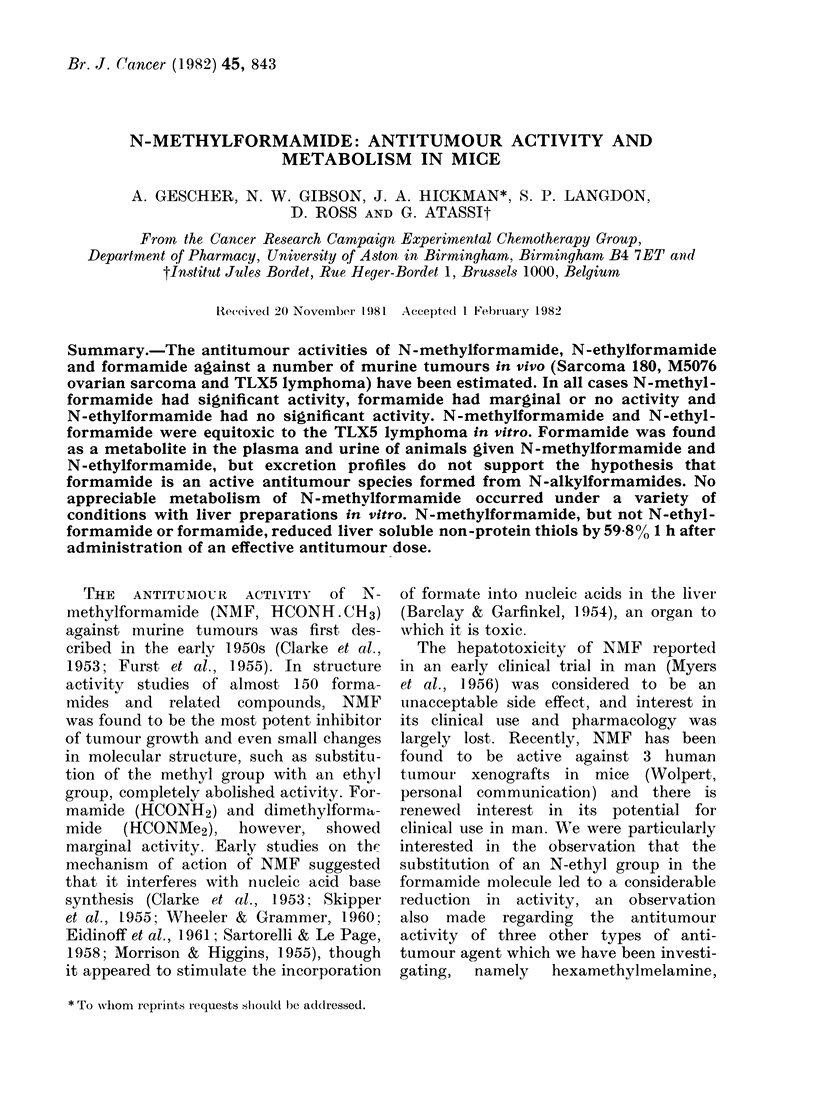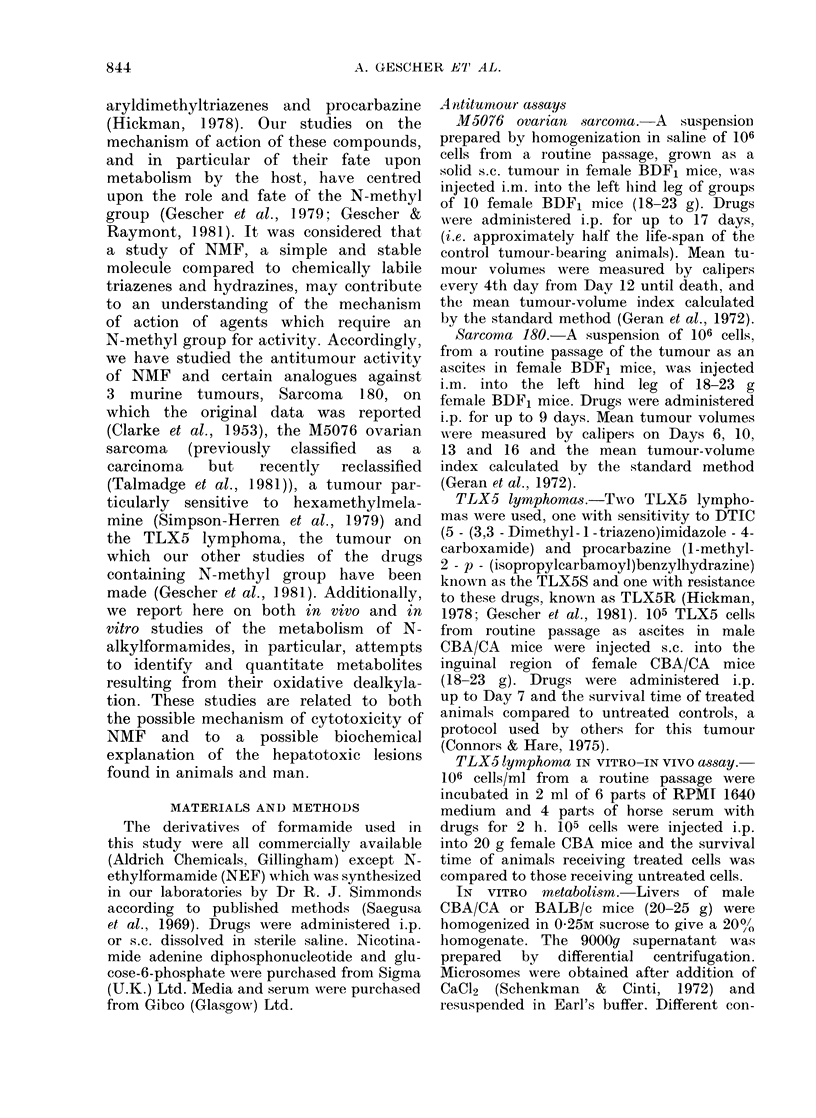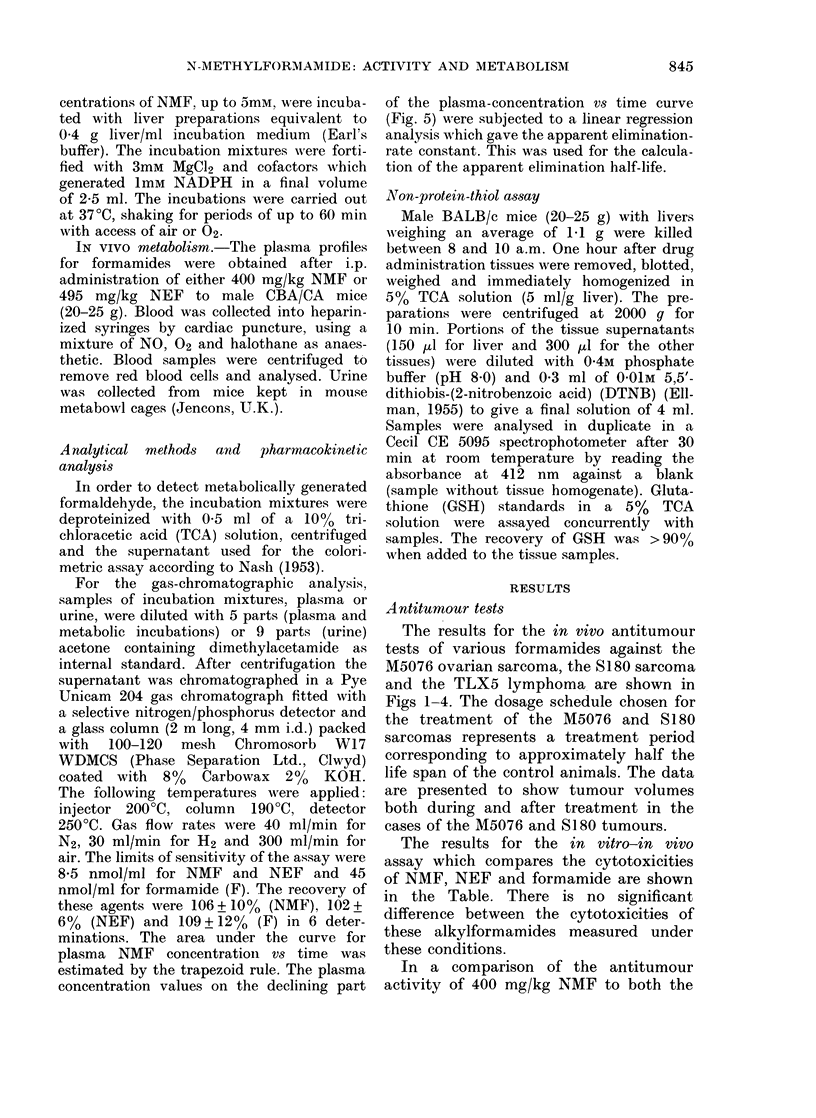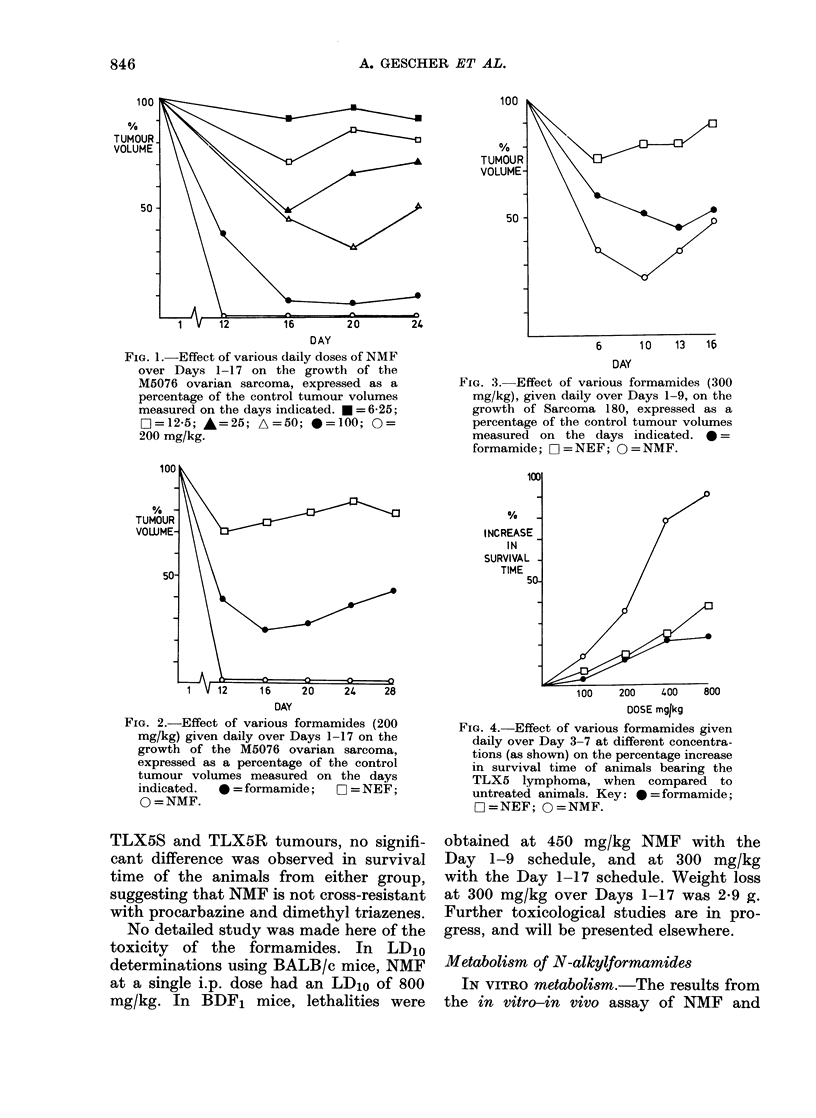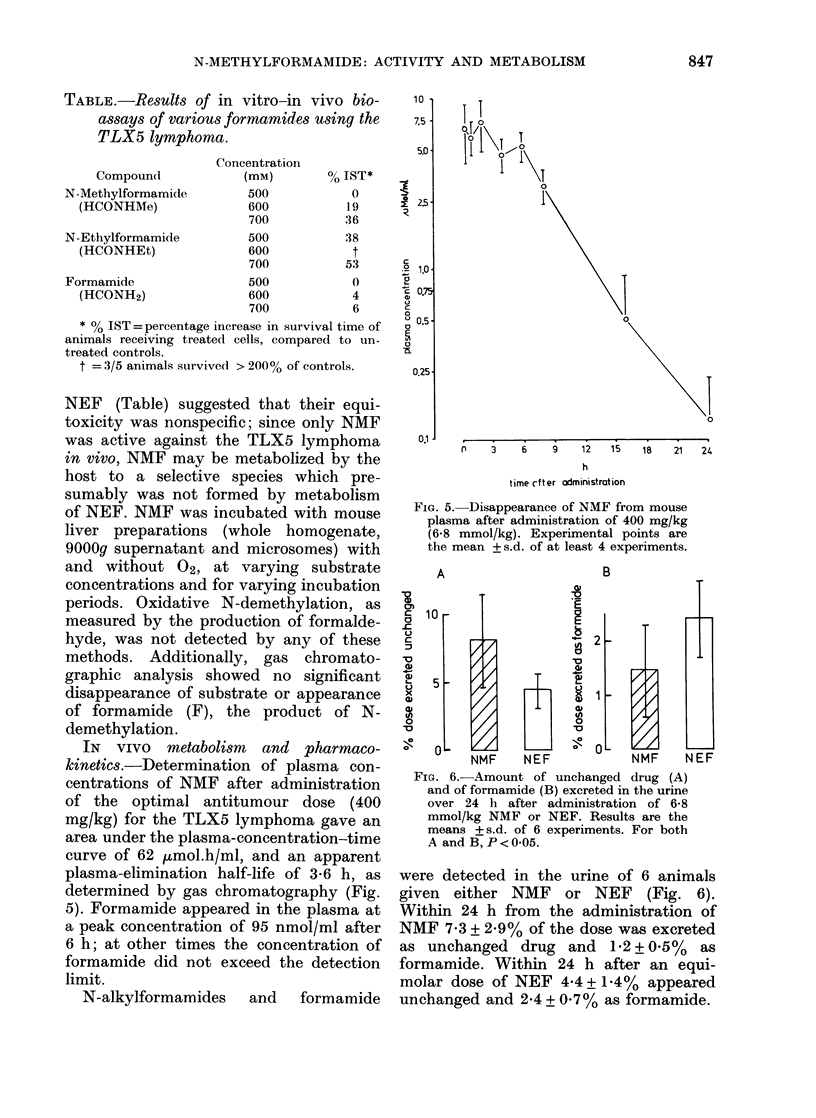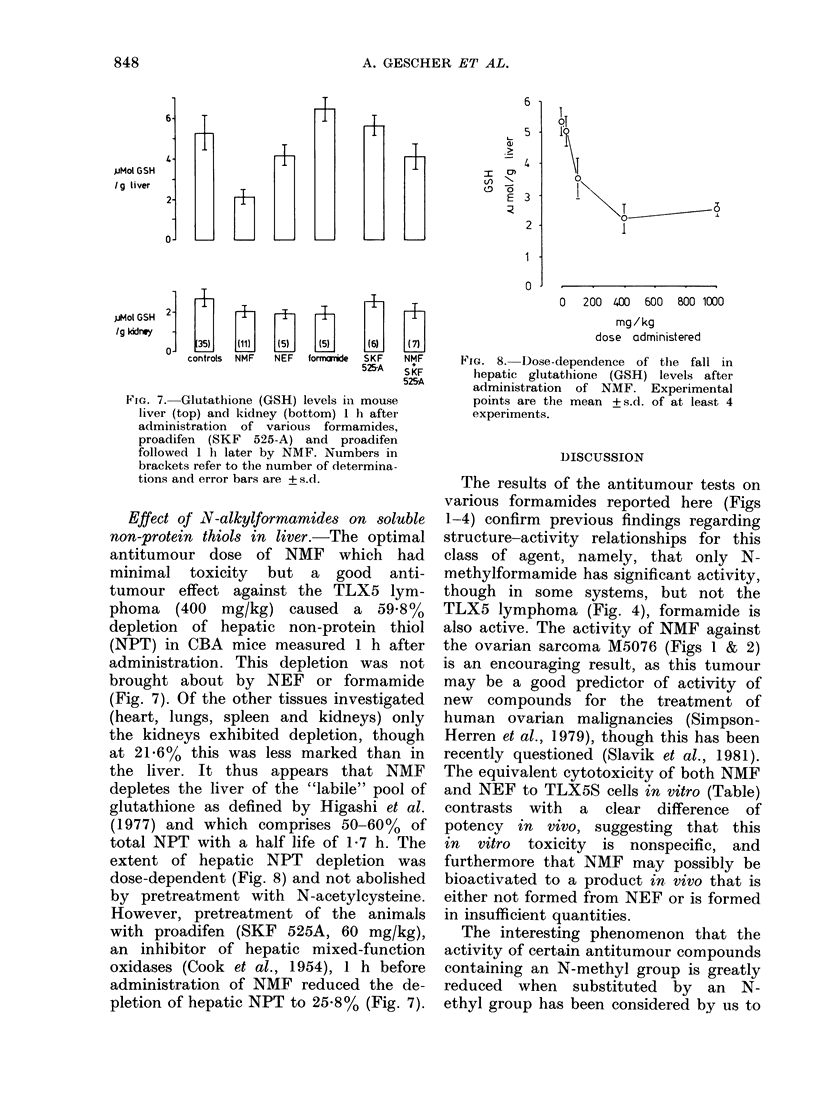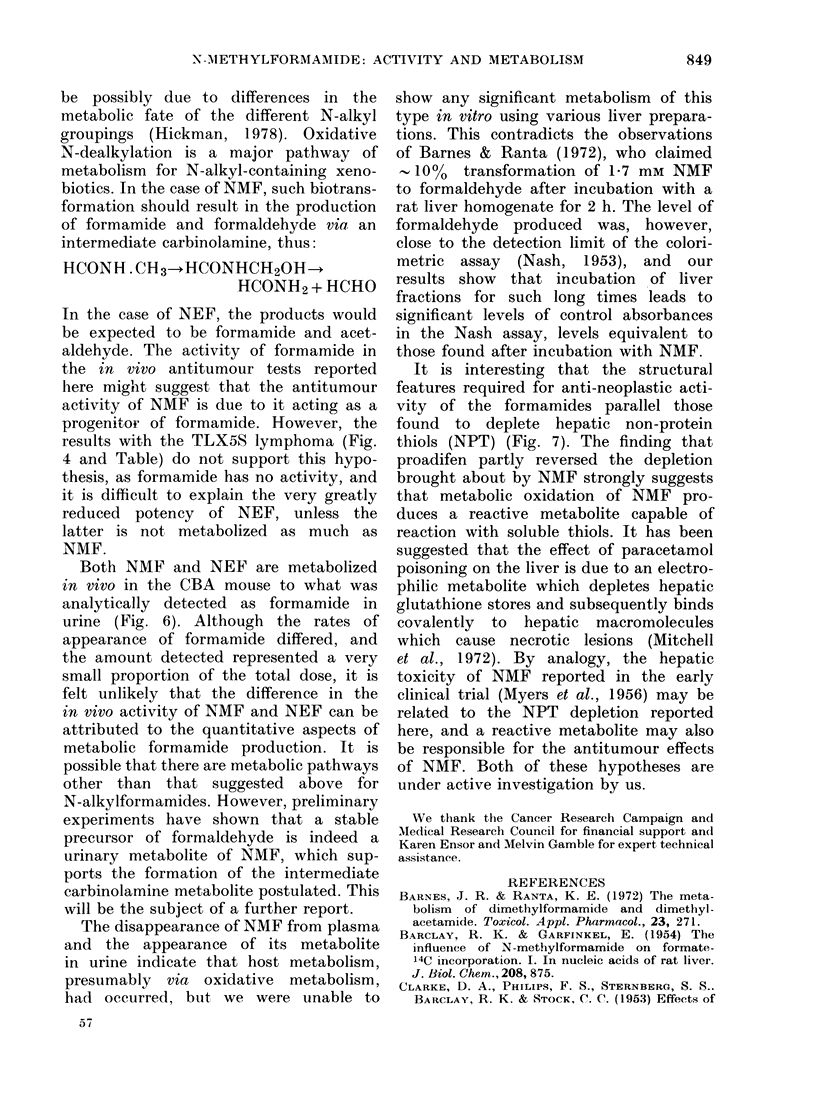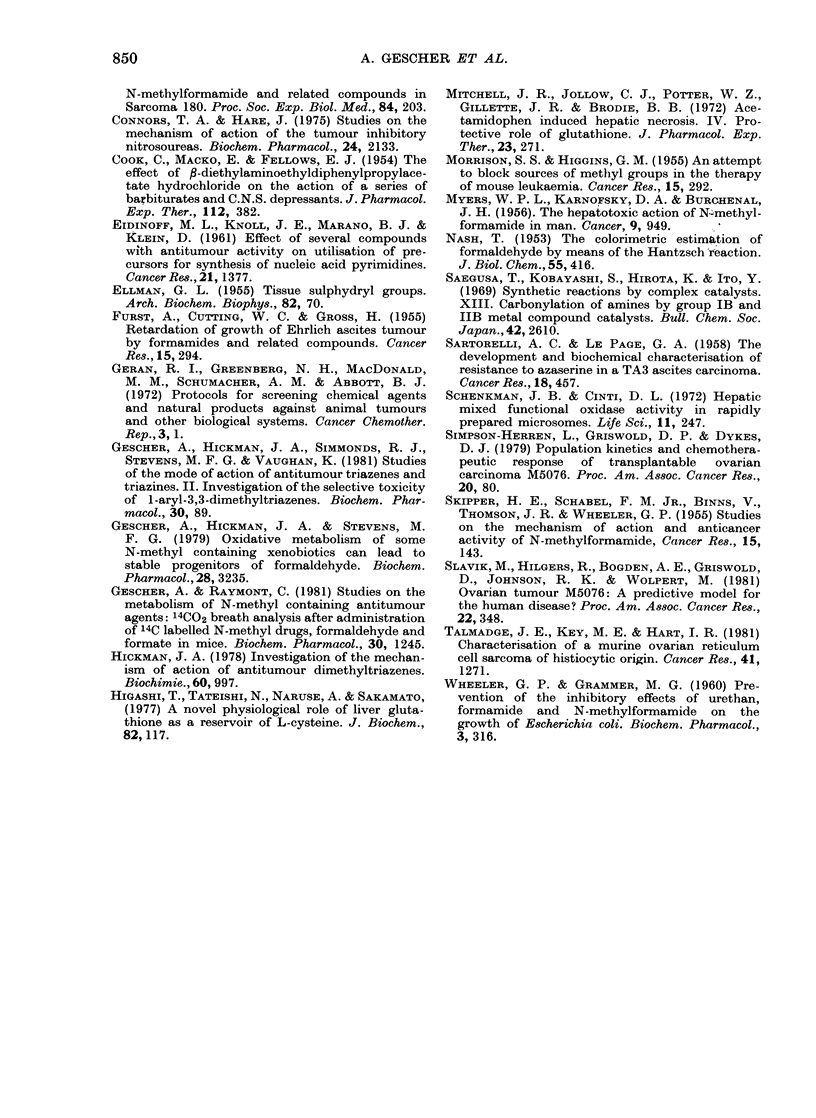# N-methylformamide: antitumour activity and metabolism in mice.

**DOI:** 10.1038/bjc.1982.136

**Published:** 1982-06

**Authors:** A. Gescher, N. W. Gibson, J. A. Hickman, S. P. Langdon, D. Ross, G. Atassi

## Abstract

The antitumour activities of N-methylformamide, N-ethylformamide and formamide against a number of murine tumours in vivo (Sarcoma 180, M5076 ovarian sarcoma and TLX5 lymphoma) have been estimated. In all cases N-methyl-formamide had significant activity, formamide had marginal or no activity and N-ethylformamide had no significant activity. N-methylformamide and N-ethylformamide were equitoxic to the TLX5 lymphoma in vitro. Formamide was found as a metabolite in the plasma and urine of animals given N-methylformamide and N-ethylformamide, but excretion profiles do not support the hypothesis that formamide is an active antitumour species formed from N-alkylformamides. No appreciable metabolism of N-methylformamide occurred under a variety of conditions with liver preparations in vitro. N-methylformamide, but not N-ethylformamide or formamide, reduced liver soluble non-protein thiols by 59.8% 1 h after administration of an effective antitumour dose.


Br. J. Cancer (1982) 45, 843

### N-METHYLFORMAMIDE: ANTITUMOUR ACTIVITY AND METABOLISM IN MICE

A. GESCHER, N. W. GIBSON, J. A. HICKMAN*, S. P. LANGDON, D. ROSS AND G. ATASSI†

*From the Cancer Research Campaign Experimental Chemotherapy Group, Department of Pharmacy, University of Aston in Birmingham, Birmingham B4 7ET and †Institut Jules Bordet, Rue Heger-Bordet 1, Brussels 1000, Belgium*

Received 20 November 1981 Accepted 1 February 1982

**Summary.—The antitumour activities of N-methylformamide, N-ethylformamide and formamide against a number of murine tumours *in vivo* (Sarcoma 180, M5076 ovarian sarcoma and TLX5 lymphoma) have been estimated. In all cases N-methylformamide had significant activity, formamide had marginal or no activity and N-ethylformamide had no significant activity. N-methylformamide and N-ethylformamide were equitoxic to the TLX5 lymphoma *in vitro*. Formamide was found as a metabolite in the plasma and urine of animals given N-methylformamide and N-ethylformamide, but excretion profiles do not support the hypothesis that formamide is an active antitumour species formed from N-alkylformamides. No appreciable metabolism of N-methylformamide occurred under a variety of conditions with liver preparations *in vitro*. N-methylformamide, but not N-ethylformamide or formamide, reduced liver soluble non-protein thiols by 59·8% 1 h after administration of an effective antitumour dose.**

THE ANTITUMOUR ACTIVITY of N-methylformamide (NMF, HCONH.$CH_3$) against murine tumours was first described in the early 1950s (Clarke *et al.*, 1953; Furst *et al.*, 1955). In structure activity studies of almost 150 formamides and related compounds, NMF was found to be the most potent inhibitor of tumour growth and even small changes in molecular structure, such as substitution of the methyl group with an ethyl group, completely abolished activity. Formamide ($HCONH_2$) and dimethylformamide ($HCONMe_2$), however, showed marginal activity. Early studies on the mechanism of action of NMF suggested that it interferes with nucleic acid base synthesis (Clarke *et al.*, 1953; Skipper *et al.*, 1955; Wheeler & Grammer, 1960; Eidinoff *et al.*, 1961; Sartorelli & Le Page, 1958; Morrison & Higgins, 1955), though it appeared to stimulate the incorporation of formate into nucleic acids in the liver (Barclay & Garfinkel, 1954), an organ to which it is toxic.

The hepatotoxicity of NMF reported in an early clinical trial in man (Myers *et al.*, 1956) was considered to be an unacceptable side effect, and interest in its clinical use and pharmacology was largely lost. Recently, NMF has been found to be active against 3 human tumour xenografts in mice (Wolpert, personal communication) and there is renewed interest in its potential for clinical use in man. We were particularly interested in the observation that the substitution of an N-ethyl group in the formamide molecule led to a considerable reduction in activity, an observation also made regarding the antitumour activity of three other types of antitumour agent which we have been investigating, namely hexamethylmelamine,

* To whom reprints requests should be addressed.

aryldimethyltriazenes and procarbazine (Hickman, 1978). Our studies on the mechanism of action of these compounds, and in particular of their fate upon metabolism by the host, have centred upon the role and fate of the N-methyl group (Gescher *et al.*, 1979; Gescher & Raymont, 1981). It was considered that a study of NMF, a simple and stable molecule compared to chemically labile triazenes and hydrazines, may contribute to an understanding of the mechanism of action of agents which require an N-methyl group for activity. Accordingly, we have studied the antitumour activity of NMF and certain analogues against 3 murine tumours, Sarcoma 180, on which the original data was reported (Clarke *et al.*, 1953), the M5076 ovarian sarcoma (previously classified as a carcinoma but recently reclassified (Talmadge *et al.*, 1981)), a tumour particularly sensitive to hexamethylmelamine (Simpson-Herren *et al.*, 1979) and the TLX5 lymphoma, the tumour on which our other studies of the drugs containing N-methyl group have been made (Gescher *et al.*, 1981). Additionally, we report here on both *in vivo* and *in vitro* studies of the metabolism of N-alkylformamides, in particular, attempts to identify and quantitate metabolites resulting from their oxidative dealkylation. These studies are related to both the possible mechanism of cytotoxicity of NMF and to a possible biochemical explanation of the hepatotoxic lesions found in animals and man.

### MATERIALS AND METHODS

The derivatives of formamide used in this study were all commercially available (Aldrich Chemicals, Gillingham) except N-ethylformamide (NEF) which was synthesized in our laboratories by Dr R. J. Simmonds according to published methods (Saegusa *et al.*, 1969). Drugs were administered i.p. or s.c. dissolved in sterile saline. Nicotinamide adenine diphosphonucleotide and glucose-6-phosphate were purchased from Sigma (U.K.) Ltd. Media and serum were purchased from Gibco (Glasgow) Ltd.

*Antitumour assays*

*M5076 ovarian sarcoma.*—A suspension prepared by homogenization in saline of $10^6$ cells from a routine passage, grown as a solid s.c. tumour in female $BDF_1$ mice, was injected i.m. into the left hind leg of groups of 10 female $BDF_1$ mice (18–23 g). Drugs were administered i.p. for up to 17 days, (*i.e.* approximately half the life-span of the control tumour-bearing animals). Mean tumour volumes were measured by calipers every 4th day from Day 12 until death, and the mean tumour-volume index calculated by the standard method (Geran *et al.*, 1972).

*Sarcoma 180.*—A suspension of $10^6$ cells, from a routine passage of the tumour as an ascites in female $BDF_1$ mice, was injected i.m. into the left hind leg of 18–23 g female $BDF_1$ mice. Drugs were administered i.p. for up to 9 days. Mean tumour volumes were measured by calipers on Days 6, 10, 13 and 16 and the mean tumour-volume index calculated by the standard method (Geran *et al.*, 1972).

*TLX5 lymphomas.*—Two TLX5 lymphomas were used, one with sensitivity to DTIC (5 - (3,3 - Dimethyl - 1 - triazeno)imidazole - 4-carboxamide) and procarbazine (1-methyl-2 - *p* - (isopropylcarbamoyl)benzylhydrazine) known as the TLX5S and one with resistance to these drugs, known as TLX5R (Hickman, 1978; Gescher *et al.*, 1981). $10^5$ TLX5 cells from routine passage as ascites in male CBA/CA mice were injected s.c. into the inguinal region of female CBA/CA mice (18–23 g). Drugs were administered i.p. up to Day 7 and the survival time of treated animals compared to untreated controls, a protocol used by others for this tumour (Connors & Hare, 1975).

*TLX5 lymphoma* IN VITRO–IN VIVO *assay.*—$10^6$ cells/ml from a routine passage were incubated in 2 ml of 6 parts of RPMI 1640 medium and 4 parts of horse serum with drugs for 2 h. $10^5$ cells were injected i.p. into 20 g female CBA mice and the survival time of animals receiving treated cells was compared to those receiving untreated cells.

IN VITRO *metabolism.*—Livers of male CBA/CA or BALB/c mice (20–25 g) were homogenized in 0·25M sucrose to give a 20% homogenate. The 9000*g* supernatant was prepared by differential centrifugation. Microsomes were obtained after addition of $CaCl_2$ (Schenkman & Cinti, 1972) and resuspended in Earl's buffer. Different concentrations of NMF, up to 5mM, were incubated with liver preparations equivalent to 0·4 g liver/ml incubation medium (Earl's buffer). The incubation mixtures were fortified with 3mM $MgCl_2$ and cofactors which generated 1mM NADPH in a final volume of 2·5 ml. The incubations were carried out at 37°C, shaking for periods of up to 60 min with access of air or $O_2$.

In vivo *metabolism.*—The plasma profiles for formamides were obtained after i.p. administration of either 400 mg/kg NMF or 495 mg/kg NEF to male CBA/CA mice (20–25 g). Blood was collected into heparinized syringes by cardiac puncture, using a mixture of NO, $O_2$ and halothane as anaesthetic. Blood samples were centrifuged to remove red blood cells and analysed. Urine was collected from mice kept in mouse metabowl cages (Jencons, U.K.).

### *Analytical methods and pharmacokinetic analysis*

In order to detect metabolically generated formaldehyde, the incubation mixtures were deproteinized with 0·5 ml of a 10% trichloracetic acid (TCA) solution, centrifuged and the supernatant used for the colorimetric assay according to Nash (1953).

For the gas-chromatographic analysis, samples of incubation mixtures, plasma or urine, were diluted with 5 parts (plasma and metabolic incubations) or 9 parts (urine) acetone containing dimethylacetamide as internal standard. After centrifugation the supernatant was chromatographed in a Pye Unicam 204 gas chromatograph fitted with a selective nitrogen/phosphorus detector and a glass column (2 m long, 4 mm i.d.) packed with 100–120 mesh Chromosorb W17 WDMCS (Phase Separation Ltd., Clwyd) coated with 8% Carbowax 2% KOH. The following temperatures were applied: injector 200°C, column 190°C, detector 250°C. Gas flow rates were 40 ml/min for $N_2$, 30 ml/min for $H_2$ and 300 ml/min for air. The limits of sensitivity of the assay were 8·5 nmol/ml for NMF and NEF and 45 nmol/ml for formamide (F). The recovery of these agents were $106 \pm 10\%$ (NMF), $102 \pm 6\%$ (NEF) and $109 \pm 12\%$ (F) in 6 determinations. The area under the curve for plasma NMF concentration *vs* time was estimated by the trapezoid rule. The plasma concentration values on the declining part of the plasma-concentration *vs* time curve (Fig. 5) were subjected to a linear regression analysis which gave the apparent elimination-rate constant. This was used for the calculation of the apparent elimination half-life.

### *Non-protein-thiol assay*

Male BALB/c mice (20–25 g) with livers weighing an average of 1·1 g were killed between 8 and 10 a.m. One hour after drug administration tissues were removed, blotted, weighed and immediately homogenized in 5% TCA solution (5 ml/g liver). The preparations were centrifuged at 2000 *g* for 10 min. Portions of the tissue supernatants (150 $\mu$l for liver and 300 $\mu$l for the other tissues) were diluted with 0·4M phosphate buffer (pH 8·0) and 0·3 ml of 0·01M 5,5′-dithiobis-(2-nitrobenzoic acid) (DTNB) (Ellman, 1955) to give a final solution of 4 ml. Samples were analysed in duplicate in a Cecil CE 5095 spectrophotometer after 30 min at room temperature by reading the absorbance at 412 nm against a blank (sample without tissue homogenate). Glutathione (GSH) standards in a 5% TCA solution were assayed concurrently with samples. The recovery of GSH was >90% when added to the tissue samples.

### RESULTS

### *Antitumour tests*

The results for the *in vivo* antitumour tests of various formamides against the M5076 ovarian sarcoma, the S180 sarcoma and the TLX5 lymphoma are shown in Figs 1–4. The dosage schedule chosen for the treatment of the M5076 and S180 sarcomas represents a treatment period corresponding to approximately half the life span of the control animals. The data are presented to show tumour volumes both during and after treatment in the cases of the M5076 and S180 tumours.

The results for the *in vitro–in vivo* assay which compares the cytotoxicities of NMF, NEF and formamide are shown in the Table. There is no significant difference between the cytotoxicities of these alkylformamides measured under these conditions.

In a comparison of the antitumour activity of 400 mg/kg NMF to both the

FIG. 1.—Effect of various daily doses of NMF over Days 1–17 on the growth of the M5076 ovarian sarcoma, expressed as a percentage of the control tumour volumes measured on the days indicated. ■ = 6·25; □ = 12·5; ▲ = 25; △ = 50; ● = 100; ○ = 200 mg/kg.

FIG. 3.—Effect of various formamides (300 mg/kg), given daily over Days 1–9, on the growth of Sarcoma 180, expressed as a percentage of the control tumour volumes measured on the days indicated. ● = formamide; □ = NEF; ○ = NMF.

FIG. 2.—Effect of various formamides (200 mg/kg) given daily over Days 1–17 on the growth of the M5076 ovarian sarcoma, expressed as a percentage of the control tumour volumes measured on the days indicated. ● = formamide; □ = NEF; ○ = NMF.

FIG. 4.—Effect of various formamides given daily over Day 3–7 at different concentrations (as shown) on the percentage increase in survival time of animals bearing the TLX5 lymphoma, when compared to untreated animals. Key: ● = formamide; □ = NEF; ○ = NMF.

TLX5S and TLX5R tumours, no significant difference was observed in survival time of the animals from either group, suggesting that NMF is not cross-resistant with procarbazine and dimethyl triazenes.

No detailed study was made here of the toxicity of the formamides. In $LD_{10}$ determinations using BALB/c mice, NMF at a single i.p. dose had an $LD_{10}$ of 800 mg/kg. In $BDF_1$ mice, lethalities were obtained at 450 mg/kg NMF with the Day 1–9 schedule, and at 300 mg/kg with the Day 1–17 schedule. Weight loss at 300 mg/kg over Days 1–17 was 2·9 g. Further toxicological studies are in progress, and will be presented elsewhere.

### *Metabolism of N-alkylformamides*

IN VITRO *metabolism*.—The results from the *in vitro–in vivo* assay of NMF and

TABLE.—*Results of* in vitro–in vivo *bioassays of various formamides using the TLX5 lymphoma.*

| Compound | Concentration (mM) | % IST* |
|---|---|---|
| N-Methylformamide (HCONHMe) | 500 | 0 |
| | 600 | 19 |
| | 700 | 36 |
| N-Ethylformamide (HCONHEt) | 500 | 38 |
| | 600 | † |
| | 700 | 53 |
| Formamide ($HCONH_2$) | 500 | 0 |
| | 600 | 4 |
| | 700 | 6 |

* % IST = percentage increase in survival time of animals receiving treated cells, compared to untreated controls.

† = 3/5 animals survived > 200% of controls.

NEF (Table) suggested that their equitoxicity was nonspecific; since only NMF was active against the TLX5 lymphoma *in vivo*, NMF may be metabolized by the host to a selective species which presumably was not formed by metabolism of NEF. NMF was incubated with mouse liver preparations (whole homogenate, 9000*g* supernatant and microsomes) with and without $O_2$, at varying substrate concentrations and for varying incubation periods. Oxidative N-demethylation, as measured by the production of formaldehyde, was not detected by any of these methods. Additionally, gas chromatographic analysis showed no significant disappearance of substrate or appearance of formamide (F), the product of N-demethylation.

IN VIVO *metabolism and pharmacokinetics.*—Determination of plasma concentrations of NMF after administration of the optimal antitumour dose (400 mg/kg) for the TLX5 lymphoma gave an area under the plasma-concentration–time curve of 62 μmol.h/ml, and an apparent plasma-elimination half-life of 3·6 h, as determined by gas chromatography (Fig. 5). Formamide appeared in the plasma at a peak concentration of 95 nmol/ml after 6 h; at other times the concentration of formamide did not exceed the detection limit.

N-alkylformamides and formamide

FIG. 5.—Disappearance of NMF from mouse plasma after administration of 400 mg/kg (6·8 mmol/kg). Experimental points are the mean ± s.d. of at least 4 experiments.

FIG. 6.—Amount of unchanged drug (A) and of formamide (B) excreted in the urine over 24 h after administration of 6·8 mmol/kg NMF or NEF. Results are the means ± s.d. of 6 experiments. For both A and B, $P < 0{\cdot}05$.

were detected in the urine of 6 animals given either NMF or NEF (Fig. 6). Within 24 h from the administration of NMF 7·3 ± 2·9% of the dose was excreted as unchanged drug and 1·2 ± 0·5% as formamide. Within 24 h after an equimolar dose of NEF 4·4 ± 1·4% appeared unchanged and 2·4 ± 0·7% as formamide.

FIG. 7.—Glutathione (GSH) levels in mouse liver (top) and kidney (bottom) 1 h after administration of various formamides, proadifen (SKF 525-A) and proadifen followed 1 h later by NMF. Numbers in brackets refer to the number of determinations and error bars are ±s.d.

FIG. 8.—Dose-dependence of the fall in hepatic glutathione (GSH) levels after administration of NMF. Experimental points are the mean ±s.d. of at least 4 experiments.

*Effect of N-alkylformamides on soluble non-protein thiols in liver.*—The optimal antitumour dose of NMF which had minimal toxicity but a good antitumour effect against the TLX5 lymphoma (400 mg/kg) caused a 59·8% depletion of hepatic non-protein thiol (NPT) in CBA mice measured 1 h after administration. This depletion was not brought about by NEF or formamide (Fig. 7). Of the other tissues investigated (heart, lungs, spleen and kidneys) only the kidneys exhibited depletion, though at 21·6% this was less marked than in the liver. It thus appears that NMF depletes the liver of the "labile" pool of glutathione as defined by Higashi *et al.* (1977) and which comprises 50–60% of total NPT with a half life of 1·7 h. The extent of hepatic NPT depletion was dose-dependent (Fig. 8) and not abolished by pretreatment with N-acetylcysteine. However, pretreatment of the animals with proadifen (SKF 525A, 60 mg/kg), an inhibitor of hepatic mixed-function oxidases (Cook *et al.*, 1954), 1 h before administration of NMF reduced the depletion of hepatic NPT to 25·8% (Fig. 7).

### DISCUSSION

The results of the antitumour tests on various formamides reported here (Figs 1–4) confirm previous findings regarding structure–activity relationships for this class of agent, namely, that only N-methylformamide has significant activity, though in some systems, but not the TLX5 lymphoma (Fig. 4), formamide is also active. The activity of NMF against the ovarian sarcoma M5076 (Figs 1 & 2) is an encouraging result, as this tumour may be a good predictor of activity of new compounds for the treatment of human ovarian malignancies (Simpson-Herren *et al.*, 1979), though this has been recently questioned (Slavik *et al.*, 1981). The equivalent cytotoxicity of both NMF and NEF to TLX5S cells *in vitro* (Table) contrasts with a clear difference of potency *in vivo*, suggesting that this *in vitro* toxicity is nonspecific, and furthermore that NMF may possibly be bioactivated to a product *in vivo* that is either not formed from NEF or is formed in insufficient quantities.

The interesting phenomenon that the activity of certain antitumour compounds containing an N-methyl group is greatly reduced when substituted by an N-ethyl group has been considered by us to be possibly due to differences in the metabolic fate of the different N-alkyl groupings (Hickman, 1978). Oxidative N-dealkylation is a major pathway of metabolism for N-alkyl-containing xenobiotics. In the case of NMF, such biotransformation should result in the production of formamide and formaldehyde *via* an intermediate carbinolamine, thus:

$$HCONH.CH_3 \rightarrow HCONHCH_2OH \rightarrow HCONH_2 + HCHO$$

In the case of NEF, the products would be expected to be formamide and acetaldehyde. The activity of formamide in the *in vivo* antitumour tests reported here might suggest that the antitumour activity of NMF is due to it acting as a progenitor of formamide. However, the results with the TLX5S lymphoma (Fig. 4 and Table) do not support this hypothesis, as formamide has no activity, and it is difficult to explain the very greatly reduced potency of NEF, unless the latter is not metabolized as much as NMF.

Both NMF and NEF are metabolized *in vivo* in the CBA mouse to what was analytically detected as formamide in urine (Fig. 6). Although the rates of appearance of formamide differed, and the amount detected represented a very small proportion of the total dose, it is felt unlikely that the difference in the *in vivo* activity of NMF and NEF can be attributed to the quantitative aspects of metabolic formamide production. It is possible that there are metabolic pathways other than that suggested above for N-alkylformamides. However, preliminary experiments have shown that a stable precursor of formaldehyde is indeed a urinary metabolite of NMF, which supports the formation of the intermediate carbinolamine metabolite postulated. This will be the subject of a further report.

The disappearance of NMF from plasma and the appearance of its metabolite in urine indicate that host metabolism, presumably *via* oxidative metabolism, had occurred, but we were unable to show any significant metabolism of this type *in vitro* using various liver preparations. This contradicts the observations of Barnes & Ranta (1972), who claimed ~10% transformation of 1·7 mM NMF to formaldehyde after incubation with a rat liver homogenate for 2 h. The level of formaldehyde produced was, however, close to the detection limit of the colorimetric assay (Nash, 1953), and our results show that incubation of liver fractions for such long times leads to significant levels of control absorbances in the Nash assay, levels equivalent to those found after incubation with NMF.

It is interesting that the structural features required for anti-neoplastic activity of the formamides parallel those found to deplete hepatic non-protein thiols (NPT) (Fig. 7). The finding that proadifen partly reversed the depletion brought about by NMF strongly suggests that metabolic oxidation of NMF produces a reactive metabolite capable of reaction with soluble thiols. It has been suggested that the effect of paracetamol poisoning on the liver is due to an electrophilic metabolite which depletes hepatic glutathione stores and subsequently binds covalently to hepatic macromolecules which cause necrotic lesions (Mitchell *et al.*, 1972). By analogy, the hepatic toxicity of NMF reported in the early clinical trial (Myers *et al.*, 1956) may be related to the NPT depletion reported here, and a reactive metabolite may also be responsible for the antitumour effects of NMF. Both of these hypotheses are under active investigation by us.

We thank the Cancer Research Campaign and Medical Research Council for financial support and Karen Ensor and Melvin Gamble for expert technical assistance.

REFERENCES

Barnes, J. R. & Ranta, K. E. (1972) The metabolism of dimethylformamide and dimethylacetamide. *Toxicol. Appl. Pharmacol.*, **23**, 271.

Barclay, R. K. & Garfinkel, E. (1954) The influence of N-methylformamide on formate-$^{14}C$ incorporation. I. In nucleic acids of rat liver. *J. Biol. Chem.*, **208**, 875.

Clarke, D. A., Philips, F. S., Sternberg, S. S., Barclay, R. K. & Stock, C. C. (1953) Effects of

N-methylformamide and related compounds in Sarcoma 180. *Proc. Soc. Exp. Biol. Med.*, **84**, 203.

CONNORS, T. A. & HARE, J. (1975) Studies on the mechanism of action of the tumour inhibitory nitrosoureas. *Biochem. Pharmacol.*, **24**, 2133.

COOK, C., MACKO, E. & FELLOWS, E. J. (1954) The effect of $\beta$-diethylaminoethyldiphenylpropylacetate hydrochloride on the action of a series of barbiturates and C.N.S. depressants. *J. Pharmacol. Exp. Ther.*, **112**, 382.

EIDINOFF, M. L., KNOLL, J. E., MARANO, B. J. & KLEIN, D. (1961) Effect of several compounds with antitumour activity on utilisation of precursors for synthesis of nucleic acid pyrimidines. *Cancer Res.*, **21**, 1377.

ELLMAN, G. L. (1955) Tissue sulphydryl groups. *Arch. Biochem. Biophys.*, **82**, 70.

FURST, A., CUTTING, W. C. & GROSS, H. (1955) Retardation of growth of Ehrlich ascites tumour by formamides and related compounds. *Cancer Res.*, **15**, 294.

GERAN, R. I., GREENBERG, N. H., MACDONALD, M. M., SCHUMACHER, A. M. & ABBOTT, B. J. (1972) Protocols for screening chemical agents and natural products against animal tumours and other biological systems. *Cancer Chemother. Rep.*, **3**, 1.

GESCHER, A., HICKMAN, J. A., SIMMONDS, R. J., STEVENS, M. F. G. & VAUGHAN, K. (1981) Studies of the mode of action of antitumour triazenes and triazines. II. Investigation of the selective toxicity of 1-aryl-3,3-dimethyltriazenes. *Biochem. Pharmacol.*, **30**, 89.

GESCHER, A., HICKMAN, J. A. & STEVENS, M. F. G. (1979) Oxidative metabolism of some N-methyl containing xenobiotics can lead to stable progenitors of formaldehyde. *Biochem. Pharmacol.*, **28**, 3235.

GESCHER, A. & RAYMONT, C. (1981) Studies on the metabolism of N-methyl containing antitumour agents: $^{14}CO_2$ breath analysis after administration of $^{14}C$ labelled N-methyl drugs, formaldehyde and formate in mice. *Biochem. Pharmacol.*, **30**, 1245.

HICKMAN, J. A. (1978) Investigation of the mechanism of action of antitumour dimethyltriazenes. *Biochimie.*, **60**, 997.

HIGASHI, T., TATEISHI, N., NARUSE, A. & SAKAMATO, (1977) A novel physiological role of liver glutathione as a reservoir of L-cysteine. *J. Biochem.*, **82**, 117.

MITCHELL, J. R., JOLLOW, C. J., POTTER, W. Z., GILLETTE, J. R. & BRODIE, B. B. (1972) Acetamidophen induced hepatic necrosis. IV. Protective role of glutathione. *J. Pharmacol. Exp. Ther.*, **23**, 271.

MORRISON, S. S. & HIGGINS, G. M. (1955) An attempt to block sources of methyl groups in the therapy of mouse leukaemia. *Cancer Res.*, **15**, 292.

MYERS, W. P. L., KARNOFSKY, D. A. & BURCHENAL, J. H. (1956). The hepatotoxic action of N-methylformamide in man. *Cancer*, **9**, 949.

NASH, T. (1953) The colorimetric estimation of formaldehyde by means of the Hantzsch reaction. *J. Biol. Chem.*, **55**, 416.

SAEGUSA, T., KOBAYASHI, S., HIROTA, K. & ITO, Y. (1969) Synthetic reactions by complex catalysts. XIII. Carbonylation of amines by group IB and IIB metal compound catalysts. *Bull. Chem. Soc. Japan.*, **42**, 2610.

SARTORELLI, A. C. & LE PAGE, G. A. (1958) The development and biochemical characterisation of resistance to azaserine in a TA3 ascites carcinoma. *Cancer Res.*, **18**, 457.

SCHENKMAN, J. B. & CINTI, D. L. (1972) Hepatic mixed functional oxidase activity in rapidly prepared microsomes. *Life Sci.*, **11**, 247.

SIMPSON-HERREN, L., GRISWOLD, D. P. & DYKES, D. J. (1979) Population kinetics and chemotherapeutic response of transplantable ovarian carcinoma M5076. *Proc. Am. Assoc. Cancer Res.*, **20**, 80.

SKIPPER, H. E., SCHABEL, F. M. JR., BINNS, V., THOMSON, J. R. & WHEELER, G. P. (1955) Studies on the mechanism of action and anticancer activity of N-methylformamide, *Cancer Res.*, **15**, 143.

SLAVIK, M., HILGERS, R., BOGDEN, A. E., GRISWOLD, D., JOHNSON, R. K. & WOLPERT, M. (1981) Ovarian tumour M5076: A predictive model for the human disease? *Proc. Am. Assoc. Cancer Res.*, **22**, 348.

TALMADGE, J. E., KEY, M. E. & HART, I. R. (1981) Characterisation of a murine ovarian reticulum cell sarcoma of histiocytic origin. *Cancer Res.*, **41**, 1271.

WHEELER, G. P. & GRAMMER, M. G. (1960) Prevention of the inhibitory effects of urethan, formamide and N-methylformamide on the growth of *Escherichia coli*. *Biochem. Pharmacol.*, **3**, 316.